# Growth and Self-Assembly of Silicon–Silicon Carbide Nanoparticles into Hybrid Worm-Like Nanostructures at the Silicon Wafer Surface

**DOI:** 10.3390/nano8110954

**Published:** 2018-11-20

**Authors:** Manuel Alejandro Perez-Guzman, Rebeca Ortega-Amaya, Yasuhiro Matsumoto, Andres Mauricio Espinoza-Rivas, Juan Morales-Corona, Jaime Santoyo-Salazar, Mauricio Ortega-Lopez

**Affiliations:** 1 Programa de Doctorado en Nanociencias y Nanotecnología, Centro de Investigación y de Estudios Avanzados del IPN, Av. IPN 2508, Col. San Pedro Zacatenco, Ciudad de México 07360, Mexico; 2 SEES, Departamento de Ingeniería Eléctrica, Centro de Investigación y de Estudios Avanzados del IPN, Av. IPN 2508, Col. San Pedro Zacatenco, Ciudad de México 07360, Mexico; ymatsumo@cinvestav.mx (Y.M.); ortegal@cinvestav.mx (M.O.-L.); 3 Departamento de Ingeniería Eléctrica, Universidad Tecnológica de México-UNITEC MÉXICO-Campus Cuitláhuac, Norte 67 2346, Col. San Salvador Xochimanca, Ciudad de México 02870, Mexico; mauricio.inu@gmail.com; 4 Departamento de Física, Universidad Autónoma Metropolitana, Unidad Iztapalapa, Av. San Rafael Atlixco 186, Col. Vicentina, Ciudad de México 09340, Mexico; jmor@xanum.uam.mx; 5 Departamento de Física, Centro de Investigación y de Estudios Avanzados del IPN, Av. IPN 2508, Col. San Pedro Zacatenco, Ciudad de México 07360, Mexico; jsantoyo@fis.cinvestav.mx

**Keywords:** silicon, silicon carbide, nanoparticles, nanowires, graphene oxide, self-assembly, thermal reduction

## Abstract

This work describes the growth of silicon–silicon carbide nanoparticles (Si–SiC) and their self-assembly into worm-like 1D hybrid nanostructures at the interface of graphene oxide/silicon wafer (GO/Si) under Ar atmosphere at 1000 °C. Depending on GO film thickness, spread silicon nanoparticles apparently develop on GO layers, or GO-embedded Si–SiC nanoparticles self-assembled into some-micrometers-long worm-like nanowires. It was found that the nanoarrays show that carbon–silicon-based nanowires (CSNW) are standing on the Si wafer. It was assumed that Si nanoparticles originated from melted Si at the Si wafer surface and GO-induced nucleation. Additionally, a mechanism for the formation of CSNW is proposed.

## 1. Introduction

Nanostructures of organic and inorganic materials display singular physicochemical properties that depend on their chemical composition and morphology [1,2,3]. Recent advances on synthesis methods, and morphological and phase composition control [4,5] have led to the development of novel multifunctional nanomaterials for applications in medicine [6], energy conversion and storage [7], environmental remediation [8], and electronics [9].

The synthesis of the core–shell hybrid inorganic–organic nanostructures is intensively pursued because it combines both the inorganic core functionality and its stability under operation; moreover, the core itself may be designed to allow for an additional functionalization, expanding its technological applications [10,11]. In the last years, a number of graphene or graphene oxide (GO)-coated transition metal oxides [12] or chalcogenides [13] have been synthesized and tested as catalysts, drug-nanocarriers, and for energy conversion and storage [14,15].

Silicon, silicon carbide, and silicon oxide nanoparticles have been reported to be promising materials in the areas of energy conversion, sensors, catalysis, and nanomedicine [16,17,18,19,20,21]. To date, a number of physical and chemical techniques have been reported for growing silicon nanoparticles, including pulsed laser ablation, plasma processing, ball milling, chemical vapor deposition, colloidal routes, and electrochemical etching [22]. In almost all of these methods, the used raw materials were bulk silicon or highly toxic Si precursors. By using silicon on insulator (SOI) film as substrate, Zywietz et al. [23] prepared silicon nanoparticles by the pulsed laser technique. In their experiments, a pulsed laser beam was focused on the surface substrate to produce a local melting, from which liquid silicon drops were formed. They proposed a mechanism for explaining the drop formation and its transformation into 160 nm-sized solid Si nanoparticles. Similarly, amorphous 3 µm Si microparticles were obtained by Garin et al. [24] using low pressure chemical vapor deposition (CVD) at temperature between 400 °C and 600 °C, using disilane as the Si source. In this case, disilane decomposed into a variety of H–Si species in the reactor bulk to reach supersaturation, and then nucleation occurred, followed by the growth of Si nanoparticles, which were collected on the polished surface of a Si (100) n-type wafer. Shavel et al. [25] produced 4–6 nm Si nanoparticles by a colloidal route using a mixture of AlCl_3_–NaCl as molten salt solvent, some stabilizer, and silicon alkoxide precursor as (3-aminopropyl)triethoxysilane (APTES) or tetramethyl orthosilicate (TMOS). The synthesis was done at atmospheric pressure and 250 °C under Ar atmosphere.

On the other hand, composites of silicon nanoparticles embedded into carbon-derived materials are currently studied as the anode material of Li-ion batteries [26]. These nanocomposites have been prepared by electrospinning, and hydrothermal and CVD methods, and have been demonstrated to produce high-performance anodes in Li-ion battery research [27,28,29]. However, some degradation-related problems were detected, because nanosized silicon pulverizes, due to the stress promoted by the anode volume change during the cycling lithiation–delithiation processes [30]. Beyond the abovementioned ones, other applications for Si nanoparticles include solar cell manufacture [31] and biomarkers [32].

A promised application niche for silicon–carbon hybrid nanocomposites are as biological markers, since graphene oxide and silicon have a low toxicity compared with capped cysteamine or mercaptoundecanoic acid CdSe and CdTe quantum dots [33].

To our best knowledge, there are a few reports on the synthesis of carbon–silicon hybrid 1D nanocomposites. For instance, Chen et al. [28] reported the fabrication of embedded-silicon carbon nanowires, starting from Si nanoparticles that were first oxidized to form SiO_2_–Si core–shell nanostructures. The obtained nanoparticles were dispersed in Pluronic F127, polyacrylonitrile, and *N*,*N*-dimethylformamide mixtures, and then electrospun into hybrid nanofibers having Si–SiO_2_ nanoparticles, and the SiO_2_ layer was then removed with HF.

This work reports a novel method to synthesize polycrystalline Si–SiC nanoparticles on a GO-coated silicon wafer surface. The present work derives from our previous research on the synthesis of graphite-encapsulated copper or copper oxide hybrid nanoparticles at the GO film/copper foil interface, which was formed by dripping water dispersed-GO on a copper foil surface. Here, it was observed that GO promotes the growth of Cu-based nanostructures at temperatures as low as 80 °C, and that their phase and morphology strongly depended on the processing temperature [34,35]. Based on these findings and considering that a solid melts starting from its surface, at temperatures below its nominal bulk melting point, we carried out experiments to produce graphite-encapsulated silicon nanoparticles at the surface of a silicon wafer by following a similar method as that used for making the graphite-coated copper-based nanoparticles.

## 2. Materials and Methods

### 2.1. Materials

Graphite flakes (+100 mesh), sodium nitrate (≥99%), potassium permanganate (≥99%), and hexane (≥99%) were obtained from Sigma-Aldrich (Toluca, Mexico). Sulfuric acid (95–98%) was purchased from Reproquifin (Mexico City, Mexico).Hydrogen peroxide (30%) and acetone (99.77%) were obtained from J.T. Baker (Mexico City, Mexico). Ethanol (99.5%) was purchased from Reasol (Mexico City, Mexico). All reagents were used as received without further purification.

### 2.2. Preparation of the CSNW

GO was prepared from natural graphite flakes by the modified Hummers’ method, as previously reported by the authors [35]. A 1 × 1 cm^2^ p-type silicon (Si) (100)-oriented wafer was used as the substrate. Before the GO film deposition, it was degreased by sequential sonication steps in hexane, acetone, and ethanol. GO films of different thickness were deposited by dripping ~0.02 mg mL^−1^ or ~2 mg mL^−1^ GO concentrated dispersions, to obtain samples named A and B, respectively. Finally, the samples were dried at 80 °C under nitrogen flux for 15 min to consolidate a film. The GO/Si samples were annealed in a quartz tube furnace at 1000 °C for 1 h under Ar atmosphere.

### 2.3. Characterization

The structural change of GO-silicon nanocomposites was assessed by Raman Spectroscopy using a WITec alpha 300 RA+ apparatus (WITec GmbH, Ulm, Germany), with an attached 50× objective lens and 532 nm laser excitation, recorded from deposited GO and reduced graphene oxide (rGO) films. Fourier transform infrared spectroscopy (FT-IR) was done on KBr-supported sample pellets using a Perkin-Elmer spectrum 100 (Perkin-Elmer Inc., Waltham, MA, USA). Film thickness was measured in a KLA-Tencor P-15 profilometer (KLA-Tencor Corp., Milpitas, CA, USA). The morphology was studied using a field-effect scanning electron microscope (FE-SEM) Zeiss Auriga (Carl Zeiss Microscopy GmbH., Jena, Germany) or Hitachi STEM-5500 (Hitachi Ltd., Tokio, Japan), and high-resolution transmission electron microscopes (HR-TEM) JEOL ARM 200F and JEOL 2010 (JEOL Ltd., Tokio, Japan).

## 3. Results and Discussion

### 3.1. Raman and FT-IR Characterization

According to Raman analysis, thermal processing at 1000 °C under an Ar atmosphere produced the simultaneous formation of the silicon nanoparticles or carbon–silicon nanowires and GO reduction. Similar results regarding the reduction level and the silicon–silicon carbide nanoparticles formation were obtained for both samples, A and B.

**Figure 1:**
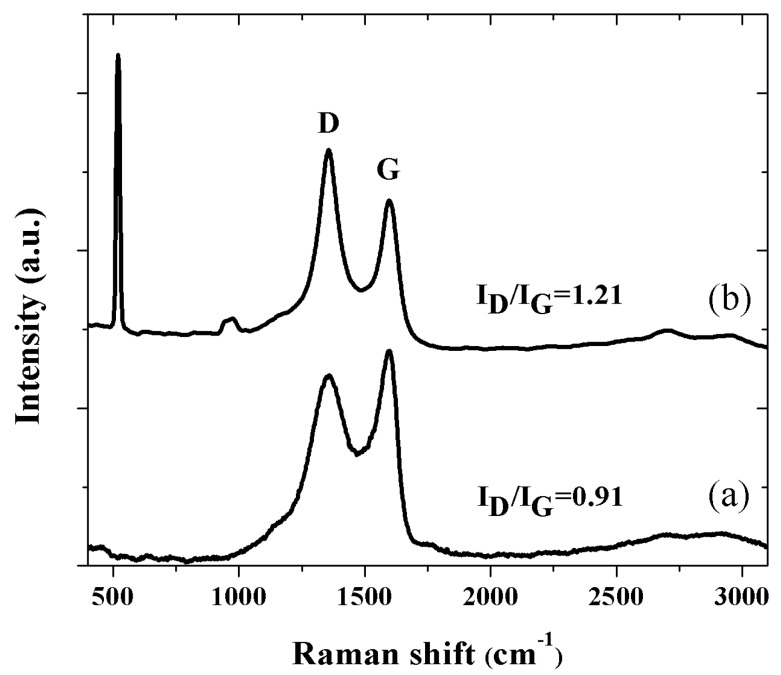
Raman spectra of as-deposited graphene oxide (GO) (**a**) and reduced graphene oxide (rGO)-Si hybrid nanocomposite (**b**). The latter displays Raman bands of Si (namely, the substrate) at 515 cm^−1^ and nanosized Si at 950 cm^−1^.

Figure 1 shows Raman spectrum (sample B) of (a) as-deposited GO and (b) one corresponding to GO–Si hybrid nanocomposite on the Si substrate. The as-deposited GO Raman spectrum exhibits the G and D bands at 1598 and 1357 cm^−1^, respectively. As frequently reported, they originate from the in-plane vibrations of carbon sp^2^ bonds (G band), and structural defects (D band) [36,37]. After the thermal process (Figure 1b), both bands changed in position and intensity, consistently with the GO reduction degree [38]. That is, the G and D peaks underwent a red-shift of about 13 and 10 cm^−1^, respectively. The red shift of the G band suggests the reduction of graphene oxide and the recovery of the sp^2^ domain, while the D band shift is associated with the size of the in-plane sp^2^ domain [37,39]. Likewise, the increase in the I(D)/I(G) ratio from 0.91 to 1.21 indicates the formation of numerous aromatic domains of smaller overall size in graphene [40]. Note that Chan Lee et al. [41] reported the effective reduction of sprayed-GO films by Si.

Raman spectrum of rGO-Si nanocomposite (Figure 1b) displays the GO Raman bands and those belonging to silicon ~515 and 950 cm^−1^. The sharp band at 515 cm^−1^ could be assigned to TO vibration of Si–Si bond [42], the red shift from the assigned value of bulk silicon (~520 cm^−1^) could be attributed to the presence of nanocrystals in the nanowire [43]. The low intensity Raman band at 950 cm^−1^ appears to correspond to nanosized silicon, as reported by Meier et al. [44], however, the HR-TEM analysis of prepared material revealed that polycrystalline nanoparticles composed of Si and SiC were obtained.

**Figure 2:**
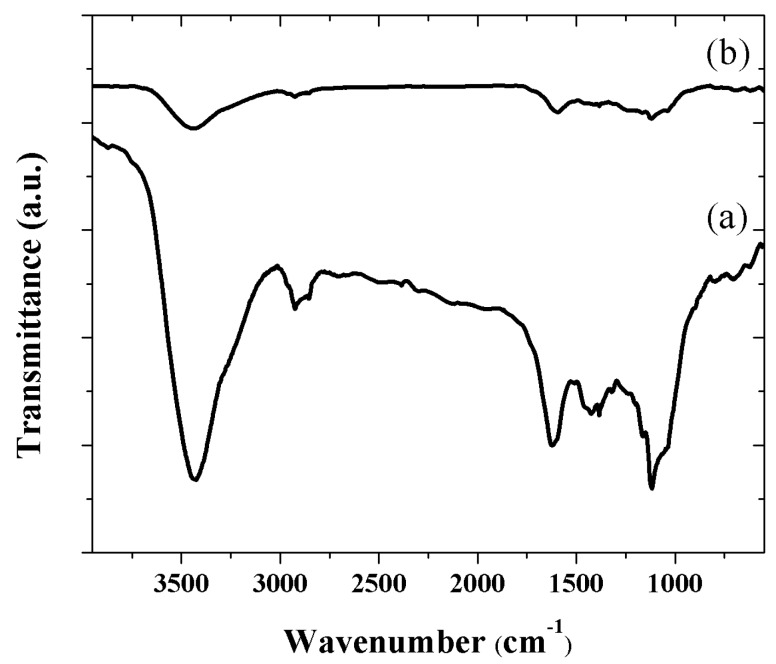
FT-IR spectra of (**a**) as-deposited GO and annealed sample at (**b**) 1000 °C for 1 h.

On the other hand, the FT-IR characterization served to confirm the effective GO reduction as indicated by Raman spectroscopy. As-deposited GO (Figure 2a) shows absorption bands at 1117, 1201, 1427, 1622, and 3425 cm^−1^, corresponding to C–O stretching vibrations, C–OH stretching vibrations, O–H deformation, C=C stretching (skeletal vibrations from unoxidized graphitic domains), and O–H stretching vibrations, respectively, all of them in good agreement with the wavenumber localization reported for GO prepared by the Hummers’ method [45,46].

The FT-IR spectrum of GO–Si nanoparticle composite (Figure 2b) also suggests the reduction of thermally processed GO with the temperature, since all the absorption bands associated with the abovementioned oxygenated groups decreased in intensity. It was even observed that the thermal process led to the entire vanishing of some bands. In the FT-IR spectra, both of the silicon nanoparticles and of the carbon and silicon nanowires, the bands associated to Si–C or Si–O cannot be appreciated in Figure 2b, since the signals are screened, due to the core–shell structure [47].

### 3.2. Morphology and Structure

**Figure 3:**
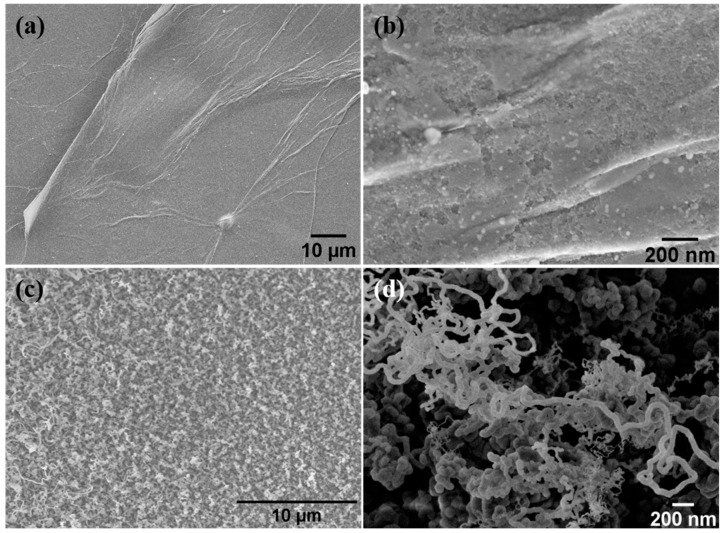
FE-SEM images of sample A (**a**,**b**) (nanoparticles decorating rGO sheets) and sample B (**c**,**d**) (the CSNW obtained from the thermal annealing of the GO at 1000 °C, using a Si wafer as support).

After being prepared, the structure and morphology of samples A and B were examined by FE-SEM and HR-TEM. It was found that the thermal annealing led to the formation of silicon-derived nanoparticles at the GO/Si interface, and their morphology and mean particle size strongly depended on the GO film thickness. The HR-TEM analysis revealed that the nanoparticles are multiphase, comprising Si and SiC as dominant phases. The sample A (GO film thickness of around 100 nm) consists of Si–SiC nanoparticles of 20 nm mean size decorating the GO sheet surface (Figure 3a,b). On the other hand, in sample B (GO film thickness of 1 μm), Si–SiC nanoparticles (30 nm mean size) were self-assembled into worm-like hybrid nanostructures, as shown in the FE-SEM images at low- and high-magnification (Figure 3c,d, respectively). These hybrid rGO-Si–SiC worm-like nanostructures are mostly 10–100 nm in diameter with a length of several micrometers, covering a relatively large area of the Si wafer surface (note the magnitude scale of FE-SEM image in Figure 3d).

**Figure 4:**
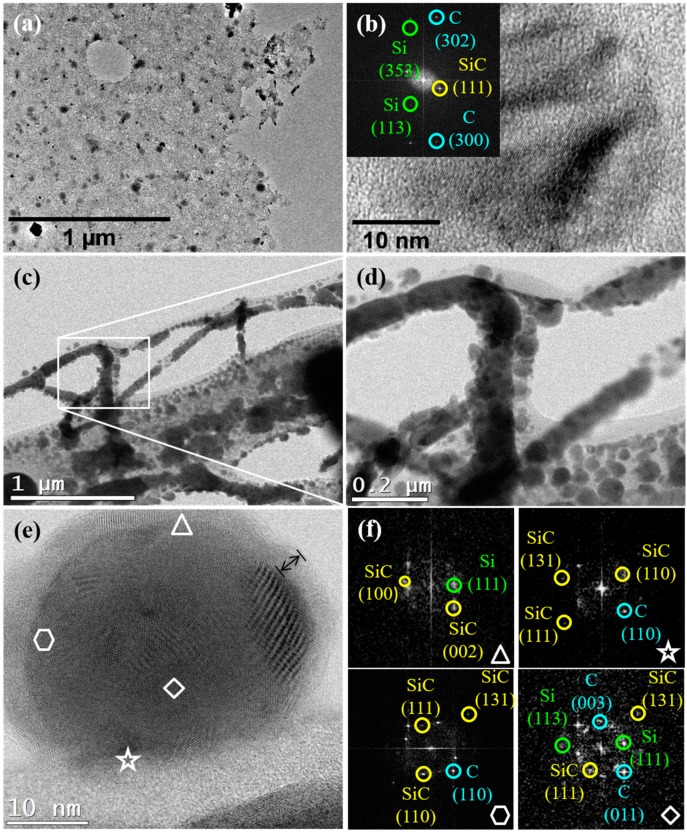
TEM and HR-TEM images of the sample A (**a**,**b**) and the sample B (**c**–**e**) after thermal annealing of GO at 1000 °C. (**a**) rGO sheets decorated by Si-based polydispersed nanoparticles. (**b**) HR-TEM of an irregularly shaped nanoparticle, where the inset corresponds to the FTT of (**b**). (**c**,**d**) Images of carbon–silicon-based nanowires (CSNW) which consist of Si-based nanoparticles embedded into 1D self-assembled rGO sheets. (**e**) HR-TEM of a nanoparticle, where it can be observed that it is polycrystalline. (**f**) The FTT of selected areas in (**e**).

The TEM and HRTEM images in Figure 4 display the morphological features of samples A and B. Figure 4a corroborates the FE-SEM characterization result in that Si-based nanoparticles decorate the rGO sheets in the sample A. On the other hand, Figure 4c,d displays the striking morphology of sample B, which consists of different-sized nanoparticles that self-assembled to develop the worm-like structures, as those shown in Figure 3d. TEM images, Figure 4c,d, suggest that silicon-derived nanoparticles are embedded into 1D self-assembled rGO sheets. In Figure 4d, a magnified image of the framed zone in Figure 4b is shown. It provides a more detailed view of the branched characteristics of the worm-like nanostructures, as they are formed by rGO-supported individual nanoparticles.

The HR-TEM images of individual nanoparticles of samples A and B are shown in Figure 4b,e, respectively. It is seen that both are 30 nm diameter ball-shaped nanoparticles, apparently coated with an rGO shell. A careful determination of interplanar spacing revealed that various crystalline phases coexist in the formed nanoparticles, that is, they are multiphase polycrystalline entities; geometric shapes in Figure 4c were used to indicate the coexisting crystalline phases.

We report fast Fourier Transform (FTT) images in the inset of Figure 4b, and the areas selected in Figure 4c were used to estimate interplanar distances, which agree well with that reported for the spacing of (100), (002), (110), (111), and (131) planes of cubic SiC, (111), (113), and (353) of Si, and (110), (011), (302), and (300) of graphite (C). Note that, if they exist, we were unable to detect silicon oxide-derived phases.

### 3.3. Mechanism Proposed for the CSNW Formation

The morphological characterization by FE-SEM and HR-TEM suggests that polycrystalline Si–SiC nanoparticles arise as the primary building blocks to develop more complex nanostructures. Both the mean particle size and morphology are strongly associated to the GO film thickness. Indeed, experiments carried out on silicon wafer GO-free surface were unsuccessful in growing silicon nanoparticles. After being formed, they interact, leading to a self-assembly process that produces the worm-like structures.

To explain the silicon nanoparticle formation, we have assumed that they originate from melted silicon at the silicon surface through a nucleation process that starts at the high energy edges of GO sheets. Since the Si melting point is around 1414 °C [48], and the hybrid materials were made at 1000 °C, we proposed that impurities and structural defects at the silicon wafer surface might promote the melting of silicon surface (pre-melting effect) at temperatures lower than the nominal silicon melting point. Also, it is quite probable that GO has a certain participation in the melting of silicon surface, because recent reports suggest that GO could act as a high temperature reactor [49]. Thus, we believe that the synergy between the pre-melting effect and the ability of GO to absorb IR radiation [50] promotes melting of the silicon surface. Additional support for the silicon surface pre-melting at temperatures lower than its melting point is provided by the roughness change observed in thermal oxidation at the silicon wafer surface, at temperatures of 900–1000 °C [51,52,53].

Regarding the phase composition of nanoparticles, it is known that the temperature of GO pyrolysis strongly depends on GO sheet size. It is quite probable that the combined participation of both GO pyrolysis and CO_2_ emission by GO reduction supply elemental carbon to react with silicon and, in so doing, form the observed silicon carbide. The formed nanoparticles are stabilized by the rGO network.

**Figure 5:**
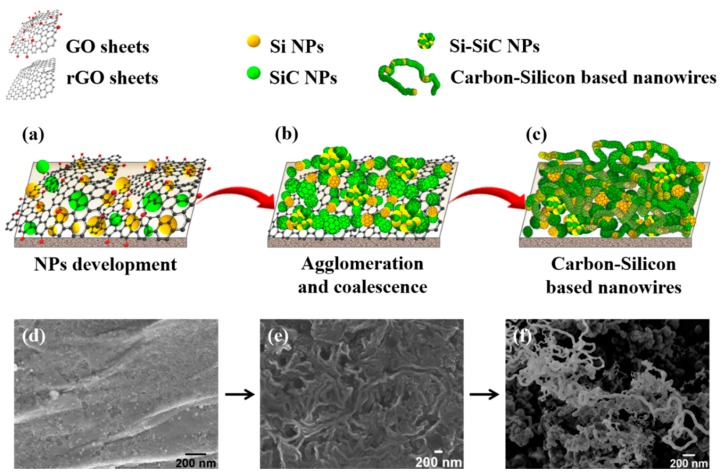
Scheme of the proposed mechanism for the carbon–silicon-based nanowires formation. (**a**) Si-based nanoparticle development at the interface GO and Si wafer. (**b**) nanoparticle agglomeration and coalescence into larger structures. (**c**) carbon-silicon based nanowires formation. FE-SEM images to support the proposed steps (**d**) development. (**e**) agglomeration and coalescence and (**f**) carbon-silicon based nanowires formation.

Accordingly, we propose that the observed worm-like hybrid nanostructures develop as illustrated in Figure 5.

At first, small Si–SiC nanoparticles develop (Figure 5a), then, they diffuse across the melted silicon surface to interact and, finally, they coalesce to form larger nanostructures (Figure 5b). The worm-like structure originates from the GO-promoted self-assembly of individual Si, SiC, and Si–SiC polycrystalline nanoparticles, with the latter formed by the coalescence among Si and SiC (see Figure 5c), as suggested by HR-TEM (Figure 4). The FE-SEM images (Figure 5d–f) were added to support the proposed mechanism for the nanoparticle self-assembly.

## 4. Conclusions

In summary, we have presented the development of silicon–silicon carbide (Si–SiC) nanoparticles at the graphene oxide/silicon interface. Depending on the GO film thickness, the obtained nanoparticles disperse on the silicon surface, or they self-assemble into a few-micrometer 1D worm-like hybrid nanostructure. It was demonstrated that dispersed silicon nanoparticles develop on graphene oxide layers, whilst 1D nanostructures comprise GO-embedded self-assembled Si–SiC nanoparticles. It was assumed that the silicon surface melts, and that Si and SiC nanoparticles originated through a graphene oxide-induced nucleation of melted silicon. In addition, a mechanism for the formation of the (CSNW) was proposed.
